# Linkage Mapping Study Reveals Conservative QTL and Candidate Genes for *Fusarium* Ear Rot Resistance in Maize

**DOI:** 10.3390/plants15152361

**Published:** 2026-07-31

**Authors:** Peipei Ma, Xinxiang Li, Xin Li, Shenshen Zhong, Yibing Ren, Zijian Zhou, Jianyu Wu, Tao Li, Ruiqi Li, Yufang Xu, Huiyong Zhang

**Affiliations:** 1 College of Life Sciences, Henan Agricultural University, Zhengzhou 450002, China; mapeipei2024@163.com (P.M.);; 2 National Key Laboratory of Wheat and Maize Crop Science, Synergetic Innovation Center of Henan Grain Crops, College of Agronomy, Henan Agricultural University, Zhengzhou 450002, China

**Keywords:** maize, ear rot, disease resistance, QTL, candidate gene

## Abstract

*Fusarium* ear rot (FER), caused by *Fusarium verticillioides* (*F. verticillioides*), is a major disease of maize that reduces grain yield and quality globally. However, few major loci for FER have been verified and cloned. Resistance to FER is a quantitative trait influenced by environmental conditions, and maize genotypes completely resistant to the pathogen remain unknown. To gain a comprehensive understanding of the genetic basis of natural variation in FER resistance, a recombinant inbred line (RIL) population consisting of 257 progenies was developed by crossing the resistant line BT with the susceptible line Xi502. This population was genotyped using a set of 6807 high-density single nucleotide polymorphism (SNP) markers developed in this study. As a result, a total of five QTLs were identified by linkage mapping across three years, located on five chromosomes, and explaining 4.38–13.13% of the phenotypic variation. Among these was a major QTL, *qFER5-2*. Located on chromosome 5 within the interval 185568562–185574073, *qFER5-2* explained 13.13% of the total phenotypic variance. The two candidate genes within *qFER5-2* exhibited distinct expression profiles between the BT and Xi502 inbred lines, suggesting their potential association with FER resistance. Collectively, these findings provide candidate genetic resources for further investigation and offer potentially useful materials for maize disease resistance breeding.

## 1. Introduction

Maize (*Zea mays* L.) is one of the most important crops, serving as a staple food, livestock feed, and industrial raw material [1]. However, maize production is facing numerous challenges, particularly the threat of various fungal diseases. *F. verticillioides* is one of the key pathogens causing multiple maize diseases. This pathogen can overwinter in soil, seeds, and plant residues, and can be transmitted to maize kernels via primary roots or airborne pathways [2,3], thereby leading to systemic infection of the plant. The major maize diseases caused by *F. verticillioides* include seedling blight [4,5], stalk rot [6], ear rot [7,8,9], seed rot [10], and cob rot [11]. Among them, maize ear rot has emerged as one of the key diseases affecting global maize production [12]. This disease not only occurs widely across major maize growing regions worldwide, leading to reduced yield and deteriorated grain quality, but the mycotoxins produced by the pathogenic fungi also pose a serious threat to human and animal health [13].

Common types of maize ear rot mainly include FER, *Gibberella* ear rot (GER), *Aspergillus* ear rot (AER), *Trichoderma* ear rot, and *Helminthosporium* ear rot, among others [14]. Of these, FER and GER are the two most economically important ear rot diseases in temperate regions, whereas AER is more frequently observed in tropical and subtropical areas [14]. In China, maize ear rot was first documented in 1987 [15]. Notably, the occurrence of *F. verticillioides* induced ear rot has also been reported in Yunnan Province, marking one of the early confirmed cases of this disease in the region [14]. Subsequent extensive investigations have confirmed that *F. verticillioides* has become the dominant pathogen causing maize ear rot in China, with a wide distribution across major maize-producing regions, including Northeast China, North China, the Huang-Huai-Hai Plain, and Southwest China [13,16,17]. Maize ear rot caused by *F. verticillioides* not only leads to kernel rot but also frequently causes severe rot of the cob tissue, thereby significantly reducing both yield and grain quality [11]. In field trials, Mu et al. observed that some ears showed no visible kernel rot symptoms on the surface, yet upon dissection, extensive brown necrosis was evident inside the cob. Moreover, *F. verticillioides* ear rot often results in kernel mold, shriveling, and reduced thousand-kernel weight, with yield losses ranging from 10% to 30% [18,19,20]. In addition to yield losses, the pathogen produces various mycotoxins, including fumonisins, during infection, leading to severe grain contamination [21]. Fumonisins cause neurotoxicity, hepatotoxicity, nephrotoxicity, and carcinogenicity; consumption of contaminated maize and its products can cause poisoning in humans and livestock, such as equine leukoencephalomalacia and porcine pulmonary edema [22,23]. Fumonisin contaminated maize is difficult to remediate during storage and processing, further increasing food safety risks and trade barriers [24]. Therefore, *F. verticillioides* ear rot not only causes direct economic losses in agricultural production but also poses long-term threats to animal and human health through mycotoxin accumulation [25].

However, current chemical and agronomic measures have proven largely ineffective in controlling ear rot and the associated fumonisin contamination, falling short of sustainable disease management requirements [26]. In contrast, the most effective strategy for controlling FER and minimizing fumonisin contamination is the breeding and deployment of maize varieties with genetic resistance [27]. Maize FER resistance is a quantitative trait governed by multiple genes [28]. Identification of resistance resources and mapping of associated resistance genes or quantitative trait loci (QTL) are essential foundations for disease resistance breeding. The extensive genetic variation among breeding materials, together with the pronounced environmental modulation of phenotypic expression, poses substantial challenges to the precise mapping of QTL [29]. Mapping accuracy is often confounded by population structure and the number of molecular markers, which increase uncertainty in the estimated positions and effects of QTL [30]. Therefore, identifying resistance QTL with stable expression across environments is essential for breeding. Early linkage maps, constructed primarily with SSRs, often had uneven genomic coverage and low marker saturation. In addition, small mapping populations provided limited recombination events, resulting in broad confidence intervals that contained excessive candidate genes and thus imposed major challenges for fine mapping. Nevertheless, linkage analysis has identified multiple QTL associated with FER resistance [7,31,32]. With the development of high-throughput sequencing, SNP markers, owing to their high density, throughput, and cost effectiveness, have increasingly replaced SSRs as the primary markers for QTL mapping [28,33]. In maize FER resistance studies, SNP application has significantly improved marker saturation and genome coverage, effectively enhancing QTL detection sensitivity and resolution.

Although high-density genetic maps have markedly improved QTL mapping resolution, most previously reported QTL for FER resistance reside in relatively large confidence intervals [34], hampering the fine mapping of candidate genes and their breeding application. To address this limitation, we developed a RIL population comprising 257 progenies derived from a cross between the resistant inbred line BT and the susceptible inbred line Xi502. A high-density genetic linkage map was constructed using 6807 SNP markers. In combination with phenotypic data from three-year, multi-location evaluations of ear rot resistance, linkage analysis identified five QTLs. Notably, *qFER5-2*, located on chromosome 5 within the 185568562–185574073 interval, is particularly noteworthy, as it spans only ~5.5 kb and contains merely two candidate genes, thus providing a highly favorable target for subsequent gene cloning and functional validation.

## 2. Results

### 2.1. Phenotypic Characterization and Analysis of FER Severity and FB1 Contamination

**Figure 1:**
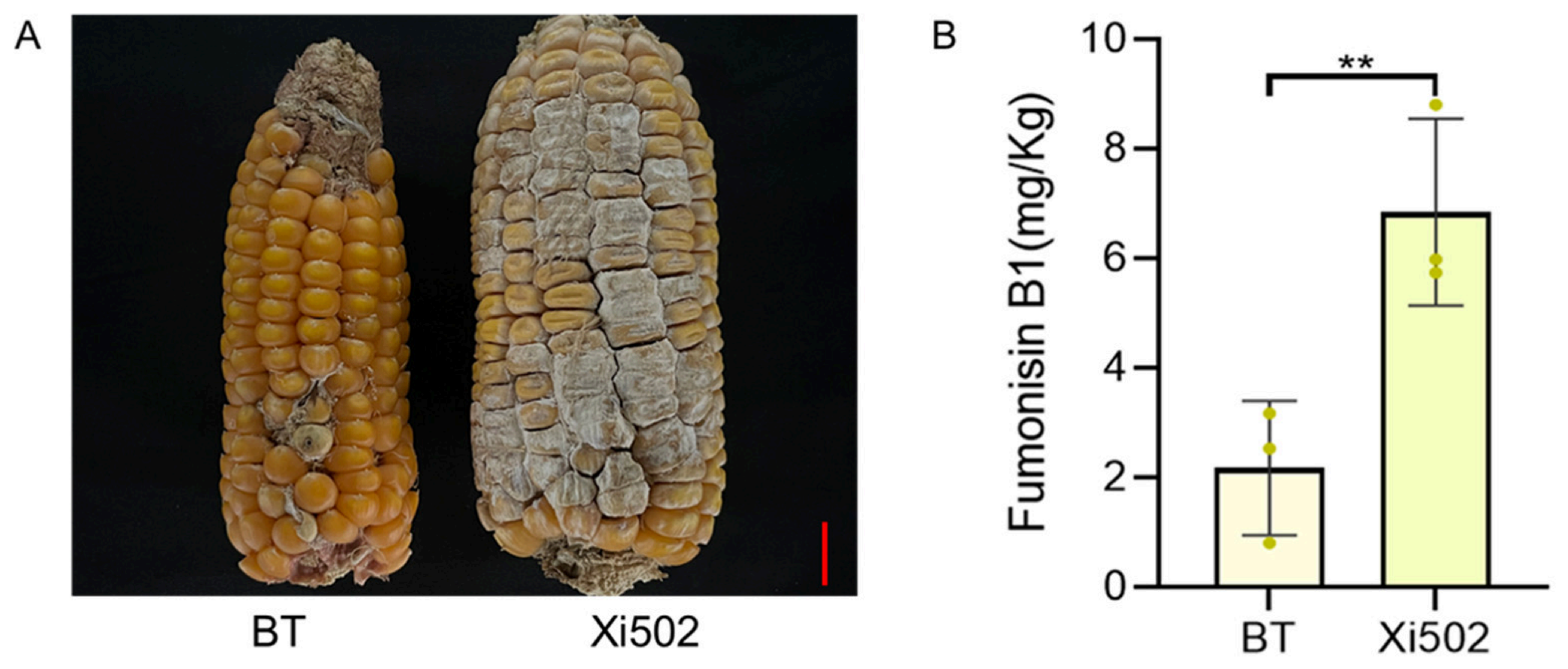
Phenotypic variation in FER severity and FB_1_ contamination at harvest among parental lines in ears artificially inoculated with *F. verticillioides*. (**A**) Photograph of ears at harvest following inoculation with *F. verticillioides* via the side-needle method at 15 days after pollination (DAP). (**B**) Five grams from each replicate were randomly sampled at 15 days after pollination for FB_1_ quantification. ** *p* < 0.01 by Student’s *t*-test.

To investigate the genetic architecture of FER resistance, we first assessed the resistant inbred line BT and the susceptible line Xi502 for disease severity. Substantial phenotypic divergence was observed between the two parents, with Xi502 consistently displaying greater disease severity and FB_1_ accumulation than BT (Figure 1). FB_1_ content was quantified via a competitive ELISA kit specific for FB_1_, using ear tissues collected from plants inoculated via the side-needle technique at 15 days after silking. Given the stable and pronounced differences between the parents, a RIL population consisting of 257 F_6_ progeny lines was constructed from the BT × Xi502 cross. This population constitutes a robust platform for the subsequent fine mapping of FER resistance and the identification of candidate QTL.

**Figure 2:**
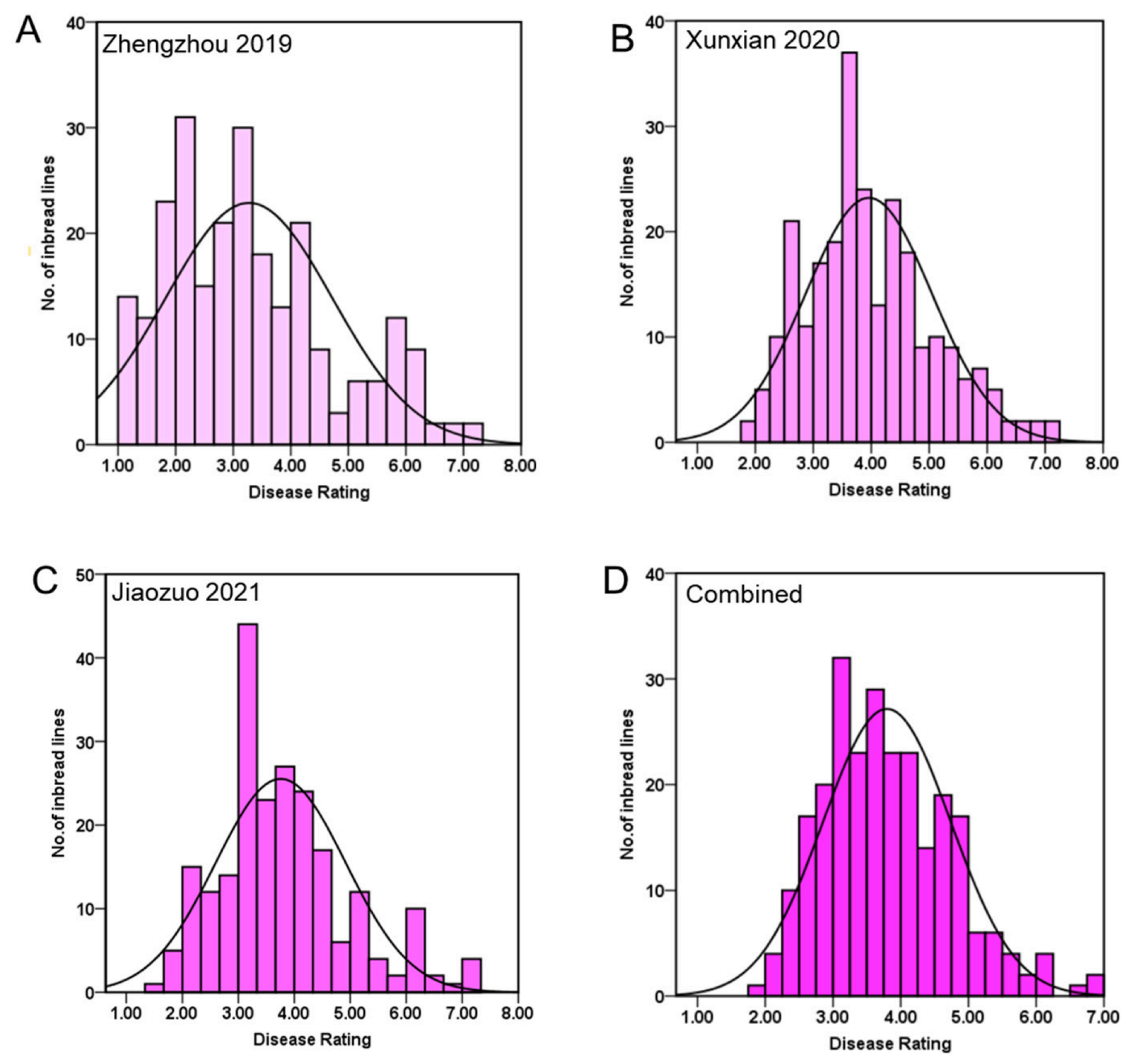
Frequency distributions of Fusarium ear rot (FER) severity among F_6_ progenies evaluated in 2019 (**A**), 2020 (**B**), and 2021 (**C**), and the BLUE (**D**) derived from side-needle inoculation assays. Normal distribution curves are overlaid for each panel.

FER resistance was assessed in the BT × Xi502 RIL population employing a standardized 1–7 visual rating scale (Table S1). Descriptive statistics for FER resistance across three environments are presented in Table 1. Mean disease severity values were consistent across environments, whereas the population exhibited wide phenotypic variation, ranging from 1.84 to 6.91, with an approximately normal distribution (Figure 2). Both genotypic variance (*σ_g_*^2^) and genotype-by-environment interaction variance (*σ_ge_*^2^) were found to be significant. Heritability (*H*^2^) was estimated at 0.71, reflecting a strong genetic component underlying the observed resistance phenotype. Collectively, these results, particularly the high heritability, support the utility of this RIL population for further genetic dissection of FER resistance.

### 2.2. Mapping of Resistance QTLs

**Figure 3:**
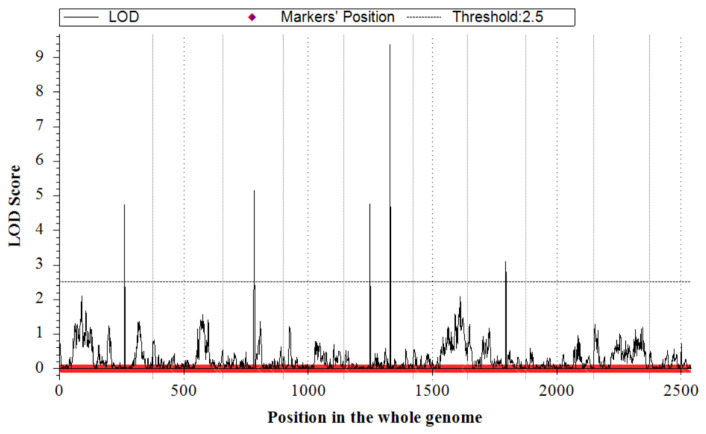
Distribution map of QTLs on chromosomes under joint analysis.

A linkage map was constructed using 6807 polymorphic SNP markers segregating between BT and Xi502, spanning all ten maize chromosomes without significant gaps (Table S2). QTL analysis was conducted based on disease index data collected from 257 RILs evaluated in three distinct environments. Five QTLs, named *qFER1*, *qFER3*, *qFER5-1*, *qFER5-2*, and *qFER7*, were detected across five chromosomes, with two loci on chromosome 5, and one each on chromosomes 1, 3, and 7 (Table 2; Figure 3). Notably, all resistance alleles were contributed by the resistant parent BT, indicating the polygenic nature of FER resistance in this population. Among these, *qFER5-2*, delimited to a ~5.5 kb interval on chromosome 5 (185568562–185574073), exhibited the highest phenotypic contribution among all detected QTL, explaining 13.13% of the total variance with a LOD score of 9.36. The narrow confidence interval and consistently significant effect across environments render *qFER5-2* a prime candidate for subsequent gene isolation and functional validation.

### 2.3. Candidate Genes at the qFER5-2 Locus

**Figure 4:**
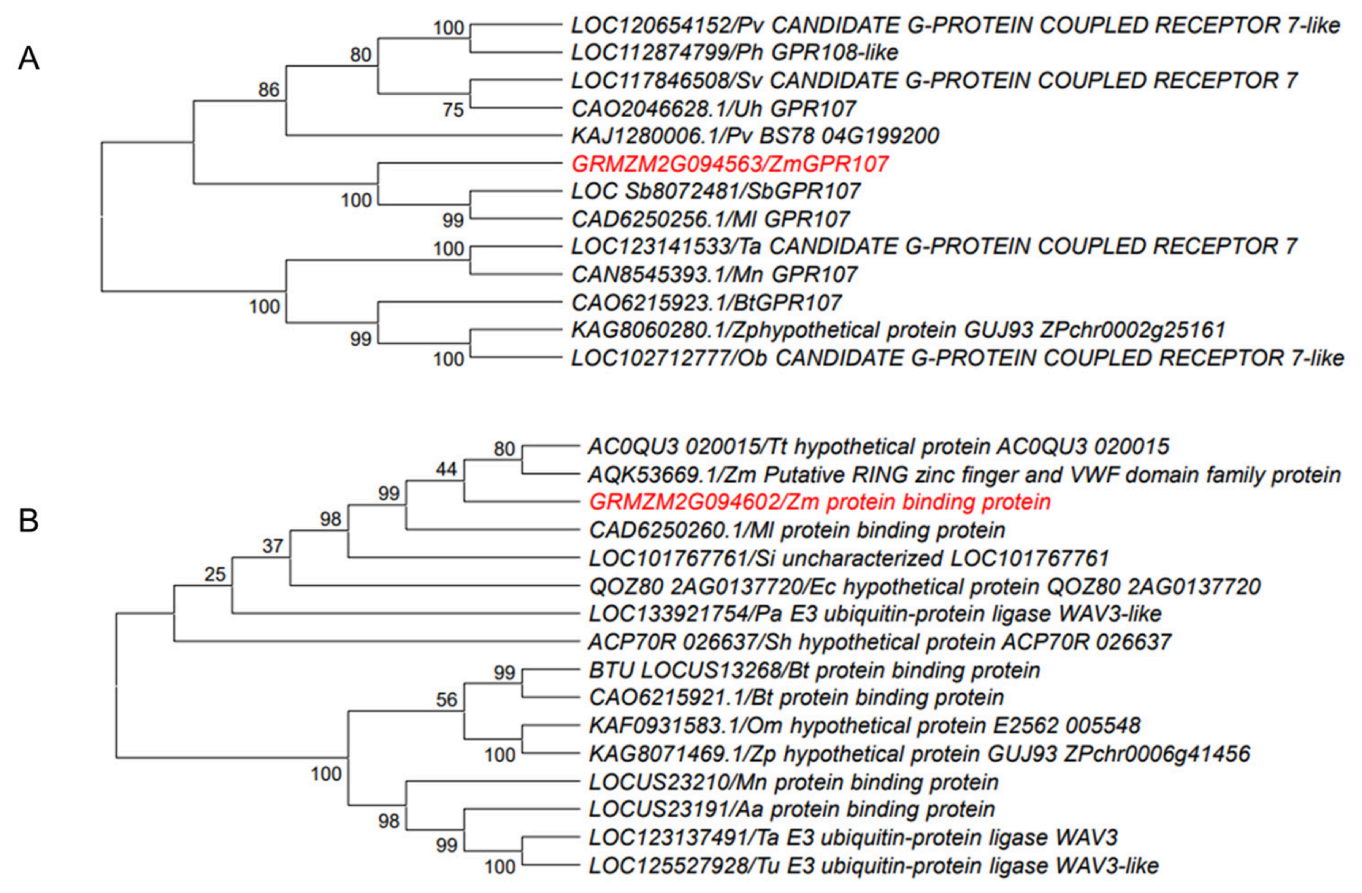
Phylogenetic analysis of candidate genes at *qFER5-2*. (**A**) Phylogenetic tree of *GRMZM2G094563* and related proteins. (**B**) Phylogenetic tree of *GRMZM2G094602* and related proteins. The target gene is highlighted in red on the phylogenetic tree.

To identify candidate genes underlying the FER resistance locus *qFER5-2*, we interrogated the physical interval spanning 185568562–185574073 on chromosome 5 using the MaizeGDB genomic database (https://www.maizegdb.org/, accessed on 5 August 2021). This analysis revealed two annotated genes: *GRMZM2G094563*, predicted to encode a lung seven-transmembrane receptor family protein, and *GRMZM2G094602*, predicted to encode a zinc finger family protein. Phylogenetic analysis demonstrated that *GRMZM2G094563* is embedded within a well-supported plant-specific clade, showing close relationships with GPR107 homologs in sorghum, wheat, and rice, which indicates its evolutionary conservation within the Poaceae family. Its close clustering with a hypothetical protein from Zea perennis further suggests possible recent gene duplication or speciation specific conservation (Figure 4A). By contrast, *GRMZM2G094602* grouped with protein binding and E3 ubiquitin ligase proteins from diverse eukaryotes, implying a conserved role in ubiquitin-mediated protein degradation or signaling pathways associated with biotic stress responses (Figure 4B). The distinct phylogenetic positions of these two genes within the narrow *qFER5-2* interval, together with their predicted protein domains, suggest that they merit further investigation as candidate loci.

**Figure 5:**
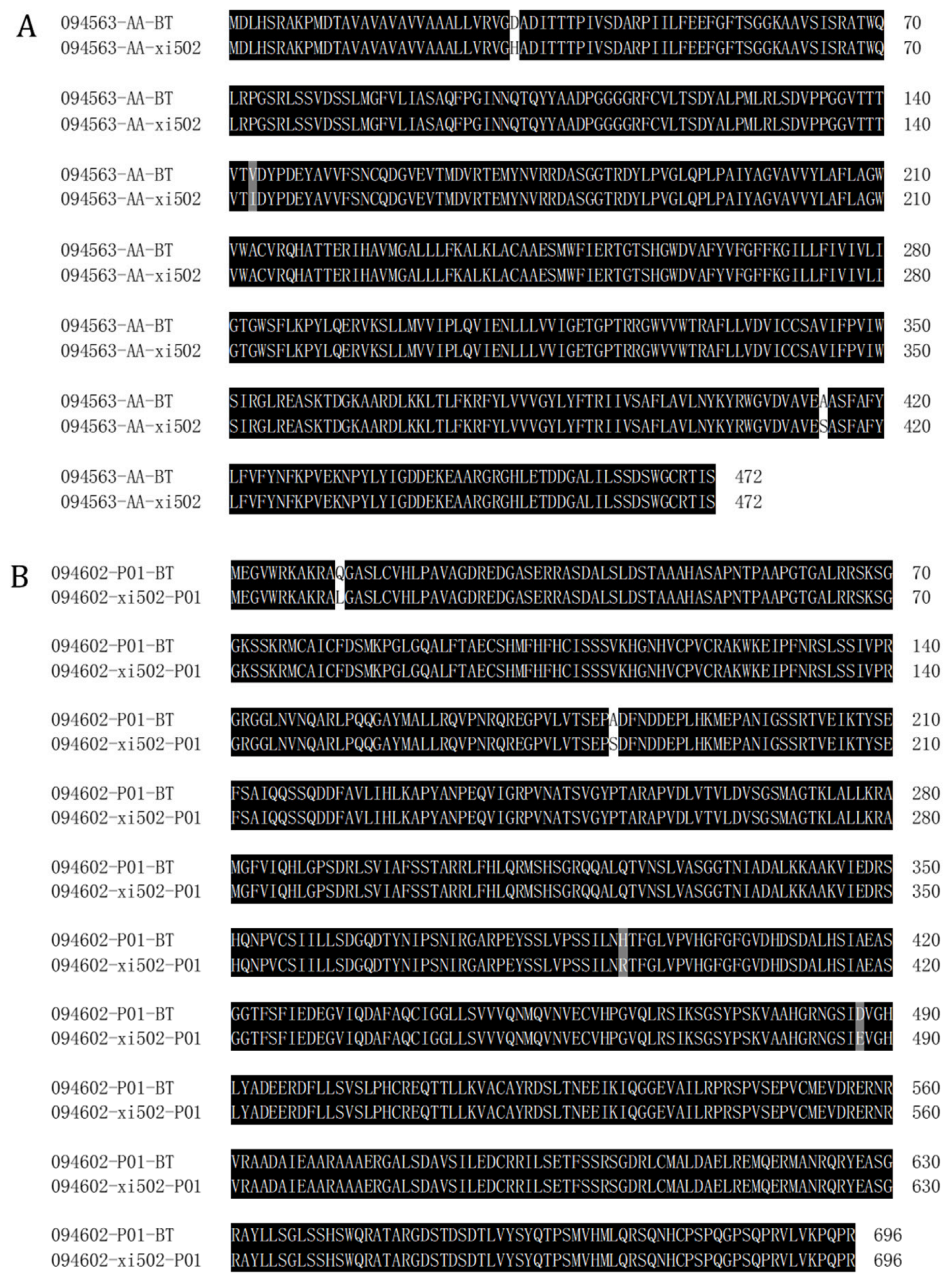
Amino acid sequence alignment of *GRMZM2G094563* (**A**) and *GRMZM2G094602* (**B**) between BT (resistant) and Xi502 (susceptible).

To investigate genomic variations within the candidate genes, the two parental lines BT and Xi502 were re-sequenced. Comparative analysis of their genomic sequences identified polymorphisms in both candidate genes. The coding sequence (CDS) of *GRMZM2G094563* is 1419 bp in length. Compared with Xi502, BT harbored three SNPs in this gene, resulting in three amino acid substitutions: aspartic acid (Asp, D) to histidine (His, H) at position 31, valine (Val, V) to isoleucine (Ile, I) at position 170, and alanine (Ala, A) to serine (Ser, S) at position 414 (Figure 5A). Notably, the substitution of a negatively charged Asp with a positively charged His at position 31 alters the electrostatic properties of this residue, which may have functional consequences. Likewise, the Ala to Ser substitution at position 414 introduces a potential phosphorylation site, as Ser residues are common targets of protein kinases; this change may enhance kinase-mediated phosphorylation at this position. The CDS of *GRMZM2G094602* is 2091 bp. Sequence comparison revealed four SNPs in BT relative to Xi502, leading to four amino acid changes: glutamine (Gln, Q) to leucine (Leu, L) at position 12, Ala to Ser at position 181, His to arginine (Arg, R) at position 392, and Asp to glutamic acid (Glu, E) at position 487 (Figure 5B). Notably, the Ala-to-Ser substitution at position 181 introduces a potential phosphorylation site, as Ser residues are common targets of protein kinases; this change may enhance kinase mediated phosphorylation at this position. Collectively, these alterations may contribute to functional divergence of the corresponding protein in the resistant parent BT.

### 2.4. Candidate Gene Expression at the qFER5-2 Locus

**Figure 6:**
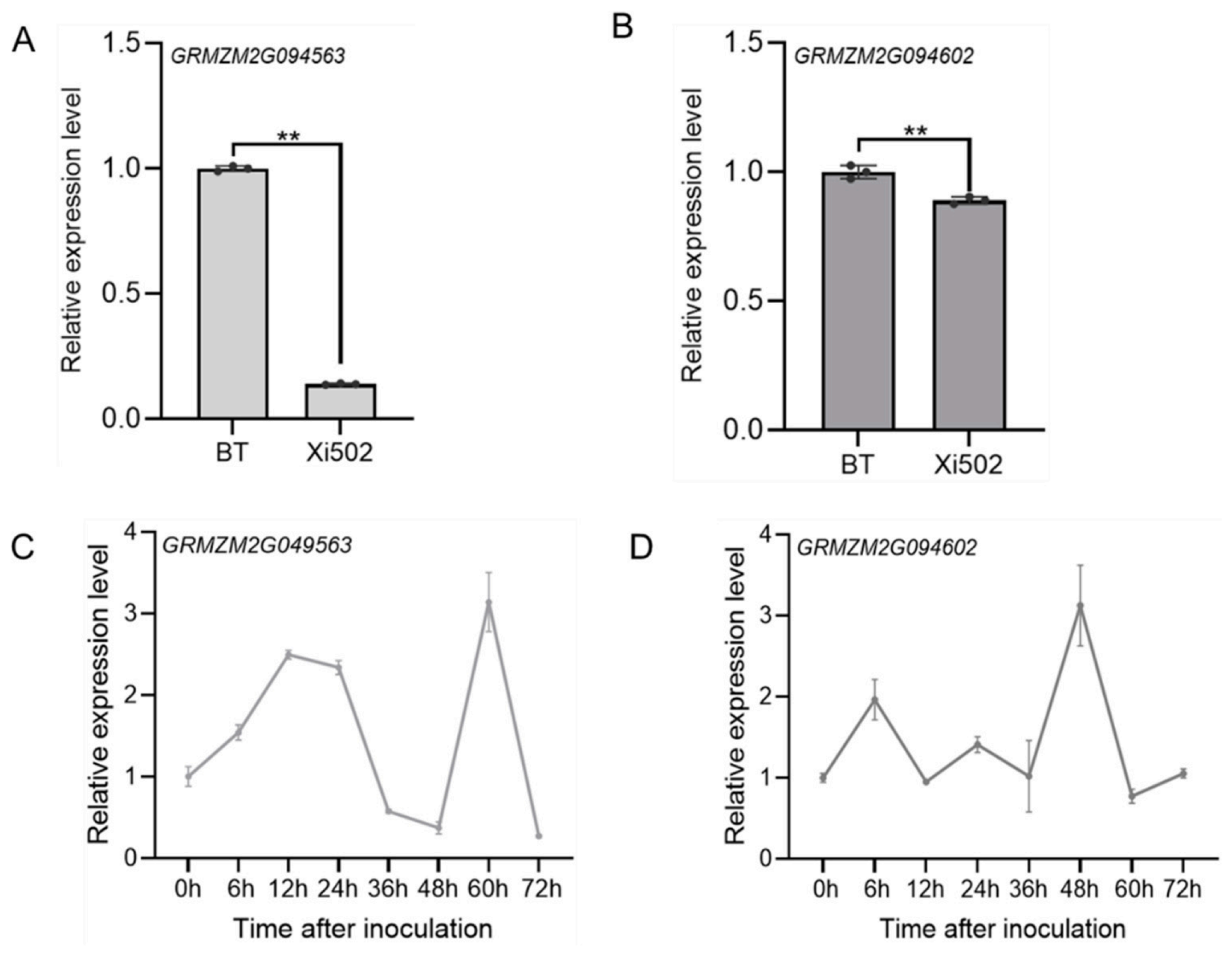
Expression of candidate genes at the *qFER5-2* locus. (**A**,**B**) Transcriptional comparison of the two candidate genes between the two parental lines. Data are shown as means ± SD of three biological replicates. (**C**,**D**) qRT-PCR of the candidate genes at different times for B73 after being inoculated with *F. verticillioides*. ** *p* < 0.01 by Student’s *t*-test.

To compare the basal expression levels of the two candidate genes between the resistant parent BT and the susceptible line Xi502, we performed quantitative real-time PCR (qRT-PCR) analysis. *GRMZM2G094563* exhibited significantly higher expression in BT relative to Xi502 (Figure 6A), suggesting a potential association between its intrinsic transcriptional activity and the differential resistance phenotypes observed between the parental genotypes. Upon inoculation with *F. verticillioides*, this gene was markedly upregulated at 12 h and 60 h post-inoculation (hpi) (Figure 6C), reflecting its rapid responsiveness to pathogen perception and implying a positive regulatory function in the host defense signaling cascade. Similarly to *GRMZM2G094563*, *GRMZM2G094602* also displayed significantly higher basal expression in BT compared with Xi502 (Figure 6B), and its transcript levels were prominently elevated upon pathogen challenge (Figure 6D).

## 3. Discussion

*F. verticillioides* is a soil- and seed-borne fungal pathogen that incites a spectrum of maize diseases, including seedling blight [4,5], stalk rot [6], ear rot [3,7,9], seed rot [10], and cob rot [11], collectively resulting in reduced grain yield, compromised quality, and health concerns. Among these, ear rot has emerged as a critical disease affecting global maize production [12]. Despite its importance, the genetic control of FER resistance remains largely unresolved, and only a few major QTLs have been cloned or functionally characterized. In the present study, we employed a RIL population derived from BT and Xi502 to dissect the genetic basis of FER resistance, and identified five QTLs via high-density SNP based linkage mapping. Of these, *qFER5-2* stood out as a major and environmentally stable QTL, explaining 13.13% of the phenotypic variance. Notably, *qFER5*-*2* was narrowed to a ~5.5 kb interval on chromosome 5 containing just two annotated genes, one of the tightest FER QTLs reported in maize, in contrast to most prior QTLs spanning hundreds of kilobases or more and harboring numerous candidates [28,33,35]. This ultra-compact interval greatly prioritizes candidate testing and reduces the complexity of resistance genetic dissection. Moreover, the major effect combined with the narrow physical span positions this QTL as a promising resource for both functional studies and breeding applications.

The stable, moderately large-effect QTL *qFER5*-*2*, consistently detected across multiple environments, is of particular interest. Unlike many previously reported QTLs with minor or environmentally unstable effects [30], *qFER5*-*2* was reliably identified over three years of field evaluation, underscoring its robustness and promising utility in marker-assisted selection (MAS). Furthermore, its physical interval (185568562–185574073) ranks among the most narrowly defined QTL regions reported for FER resistance to date [28,33,35], substantially facilitating the identification of candidate genes. The presence of only two annotated genes within this interval markedly narrows the candidate gene search, providing a focused entry point for functional studies. The two candidate genes encoding a lung seven-transmembrane receptor family protein (*GRMZM2G094563*) and a zinc finger family protein (*GRMZM2G094602*) exhibited similar expression patterns between BT and Xi502, making them noteworthy candidates for further investigation. Although their precise molecular functions remain to be elucidated, their differential regulation in response to *F. verticillioides* challenge suggests their involvement in pathogen recognition and downstream defense signaling. The physical proximity of these genes to the QTL, together with their genotype-dependent expression patterns, makes them candidates warranting further attention. To conclusively establish their roles, CRISPR/Cas9-based knockout and transgenic complementation experiments are planned as the next step in our validation pipeline.

The RIL population and high-density genetic map generated in this study provide invaluable resources for the genetic dissection of FER resistance and other traits of agronomic importance [11]. The synergy of a moderately large population, comprehensive marker coverage, and environmentally replicated phenotyping not only improves the accuracy of QTL detection but also establishes a solid groundwork for downstream fine mapping and positional cloning of candidate genes [36,37]. In addition, the resistant parent BT, which harbors multiple QTLs associated with resistance [11,38], represents a valuable donor for breeding strategies focused on pyramiding favorable alleles for enhanced and durable disease resistance. Collectively, these resources, together with the QTL identified herein, will facilitate the translation of genomic insights into breeding applications and expedite the development of FER resistant varieties.

Nonetheless, several limitations of the present study merit careful consideration. First, although the resolution of QTL mapping has been substantially enhanced, it does not yet permit direct gene cloning without recourse to additional fine mapping or independent validation populations. Second, the moderate effect size of *qFER5*-*2* (13.13% of phenotypic variance), albeit stable over years, suggests that the resistance phenotype may also be governed by minor-effect QTL or epistatic interactions. Consequently, future investigations should emphasize high-resolution fine mapping of *qFER5-2*, coupled with transcriptomic profiling and functional validation via reverse genetics, to conclusively identify the causal gene(s) and unravel the molecular basis of resistance.

## 4. Materials and Methods

### 4.1. Plant Material and Experimental Design

Two maize inbred lines with contrasting susceptibility to FER were employed in this study: BT, a resistant line originating from Thailand and designated as 8085 (Tai) [39], and Xi502, a susceptible line derived from the Tangsipingtou germplasm. To generate a segregating population, BT (♀) was crossed with Xi502 (♂), and the resulting progeny were advanced through single-seed descent to produce a RIL population comprising 257 F_6_ families. Field experiments were conducted over three growing seasons at three distinct locations in China: Zhengzhou (2019, 34°52′ N, 113°37′ E), Xunxian (2020, 35°67′ N, 114°55′ E), and Jiaozuo (2021, 35°21′ N, 113°23′ E). All sites are characterized by a temperate continental climate, with sufficient precipitation and adequate thermal and solar resources during the maize reproductive period.

At each environment, the RIL population and parental lines were arranged in a randomized complete block design with two replications. Each plot consisted of a single 2.5 m row containing ten plants, with a row-to-row spacing of 0.67 m. All plants were hand-self-pollinated, and a single insecticide application was made manually at the jointing stage to manage corn borer infestation. All other agronomic operations were carried out in accordance with locally recommended practices.

### 4.2. Inoculation and Phenotypic Evaluation

A *F. verticillioides* strain NH-1 was isolated from naturally infected maize kernels collected in Zhengzhou using the single-spore isolation method. Preliminary identification was performed based on morphological characteristics, including colony color, growth pattern, and conidial morphology, as observed under a light microscope following previously described criteria [38]. For molecular confirmation, genomic DNA was extracted from the fungal isolates, and the internal transcribed spacer (ITS) region of ribosomal DNA was amplified using the universal primers ITS1 and ITS4 [38]. The PCR products were sequenced, and BLAST analysis against the NCBI GenBank database confirmed the isolate as *F. verticillioides*. To verify the pathogenicity of NH-1, its virulence was evaluated prior to field inoculation. Ears of the susceptible maize inbred line Xi502 and the resistant line BT were inoculated with a conidial suspension (5 × 10^6^ spores mL^−1^) of the isolate. Typical disease symptoms were observed on the inoculated ears (Figure 1), confirming that NH-1 is pathogenic and suitable for resistance evaluation in the subsequent QTL mapping experiments. The fungus was propagated on sterile mature maize kernels at 28 °C in the dark for 5 days. One day prior to field inoculation, the colonized maize seed culture was resuspended in sterile water, gently stirred in one direction, and filtered through several layers of gauze into a clean beaker to obtain a spore suspension. The harvested spores were quantified using a hemocytometer and adjusted to a concentration of 5 × 10^6^ spores mL^−1^ in sterile distilled water containing 200 μL·L^−1^ Tween 80 for field inoculation.

Inoculations were performed using the needle-punch ear method, which allows precise control of the inoculum volume and is operationally simple. Three key factors were considered: timing, dosage, and inoculation site. Inoculations were carried out at 15 days after pollination, when the silks had dried and kernels exhibited moderate moisture content conditions conducive to successful infection. Inoculation timing was critical: early inoculation led to excessively severe ear rot that impaired ear development, whereas late inoculation resulted in mild symptoms and poor differentiation between resistant and susceptible lines. A volume of 1 mL of spore suspension was injected per ear, generally at the middle or slightly below the middle of the ear. For ears with poor kernel set, the inoculation point was selected at the densest kernel area. During injection, the syringe was held at an angle of less than 90 degrees relative to the ear to minimize spore suspension leakage. After inoculation, the injection site was sealed with paper tape to prevent leakage and protect against corn borer infestation, and each ear was covered with a kraft paper bag to avoid contamination by airborne pathogens.

All ears from the RIL population were harvested individually at 50 days after pollination and air-dried under natural conditions for seven days prior to phenotypic evaluation. Resistance to FER was assessed using a 1–7 rating scale described by Dong et al. [39], where 1 indicates no visible infection; 2 = 1–3% of ear surface infected; 3 = 4–10%; 4 = 11–25%; 5 = 26–50%; 6 = 51–75%; and 7 = 76–100% infection. For each line, ten ears per row were evaluated, and the disease severity score for each replication was calculated as the average of the ten ears. The mean severity score from the two replications was then used as the final disease score for each environment.

### 4.3. Measurement of Fumonisin B1 Content

Seeds of the maize lines BT and Xi502, inoculated with *F. verticillioides*, were ground to a fine powder. A 5.0 g aliquot was mixed with 25 mL of 60% (*v*/*v*) methanol and vigorously shaken for 10 min. The mixture was then centrifuged at 150 rpm for 5 min, and the resulting supernatant was collected for further analysis. FB_1_ content was quantified using a commercial competitive enzyme-linked immunosorbent assay (ELISA) kit (HUAAN MAGNECH (Beijing, China), HEM1296) specifically designed for FB_1_ detection. The assay was based on the following principle: microplate wells were pre-coated with FB_1_ antigens, which competed with FB_1_ present in the test samples for binding to FB_1_-specific antibodies. After washing, bound antibodies were detected using a secondary antibody, followed by color development with tetramethylbenzidine (TMB) substrate. Absorbance was measured at 450 nm, and the signal intensity was inversely proportional to the FB_1_ concentration in the sample. The FB_1_ content in each sample was determined by interpolating absorbance values against a standard curve and multiplying by the corresponding dilution factor.

### 4.4. Linkage Mapping

A genetic linkage map was constructed using 6807 polymorphic SNP markers that distinguished the parental lines BT and Xi502. Map construction was performed with QTL IciMapping software (version 4.2). The “rippling” command was applied iteratively to determine the optimal marker order within each linkage group. The resulting map spanned 2540.87 cM, with an average marker density of 0.37 cM per marker. Detailed information on the linkage map is provided in Supplementary Table S2. QTL associated with FER resistance were identified using the inclusive composite interval mapping (ICIM) model implemented in the QTL IciMapping software. The genome-wide significance threshold (LOD = 2.5) was established by 1000 permutation tests at α = 0.05 to control the type I error rate across the genome. For each detected QTL, the sign of the estimated additive effect indicated the direction of resistance contribution: a positive value indicated that the resistance allele was derived from Xi502, whereas a negative value indicated derivation from BT.

### 4.5. Nucleic Acid Extraction and qRT-PCR

To examine the expression patterns of candidate genes, seeds of the maize lines BT and Xi502 were surface sterilized prior to RNA extraction. Three seeds were sampled from each line, and total RNA was extracted using the TransZol Plant reagent (TRAN, Beijing, China) according to the manufacturer’s instructions. Maize elongation factor 1 alpha (*EF1α*) was selected as the endogenous reference gene. For gene expression quantification, total RNA was treated with Rnase-free DNase I and reverse-transcribed using a Transcriptor First Strand cDNA Synthesis Kit (Yeasen, Shanghai, China). The resulting cDNA was diluted 20-fold and used as a template for qRT-PCR, with *EF1α* serving as the internal control. Relative expression levels of the target genes were calculated using the 2^−ΔΔCt^ method, normalized to *EF1α* expression. Statistical significance between inoculated in B73 at each time point, as well as between resistant and susceptible parents, was assessed using a two-tailed Student’s *t*-test with unequal variances. For each genotype and treatment combination, three independent biological replicates were used, each derived from a separately grown plant. All primer sequences are listed in Supplementary Table S3.

### 4.6. Statistical Analysis

Expression data of candidate genes were analyzed using Microsoft Excel 2016. For each of the 257 RILs, FER disease index values were derived from best linear unbiased estimates (BLUEs) calculated across three environments (2019ZZ, 2020XX, and 2021JZ) using QTL IciMapping software, which was also used to estimate broad-sense heritability (*H*^2^) following the method described by Li [40]. Heritability was calculated as: *H*^2^ = *σ_g_*^2^/(*σ_g_*^2^ + *σ_ge_*^2^/*n* + *σ_e_*^2^/*nr*), where *σ_g_*^2^ is the genotypic variance, *σ_ge_*^2^ is the genotype-by-environment interaction variance, *σ_e_*^2^ is the error variance, *n* is the number of environments, and *r* is the number of replications per environment.

The frequency distribution of FER disease index was analyzed using SPSS version 19 (IBM Corp., Armonk, NY, USA). Coefficients of variation (CV) were calculated in Excel 2016 as CV = σ/μ, where σ is the standard deviation of the phenotypic mean and μ is the overall mean of the phenotype.

## 5. Conclusions

In conclusion, this study successfully mapped five QTLs for resistance to *F. verticillioides* induced ear rot using a high-density linkage map derived from a BT × Xi502 RIL population. Notably, *qFER5*-*2*, a major and environmentally stable QTL accounting for 13.13% of phenotypic variance, was delimited to a ~5.5 kb interval on chromosome 5 harboring only two candidate genes. The differential expression of these genes between resistant and susceptible parents suggests their possible association with the resistance phenotype. Collectively, these findings provide candidate genetic loci for further investigation, and the resistant line BT, together with the high-density RIL mapping population developed here, represents a useful resource for future resistance breeding and genetic studies in maize.

## Figures and Tables

**Table 1:** Descriptive statistics for FER resistance of RIL populations across environments.

	Environment	Material Number	Mean ± SD ^a^	Range ^b^	*σ_g_* ^2 c^	*σ_e_* ^2 d^	*σ_ge_* ^2 e^	*H* ^2 f^
RIL Population	In 2019	249	3.27 ± 1.45	1–7	1.36	1.33		0.64
	In 2020	254	3.97 ± 1.09	1.75–7	0.97	0.40		0.82
	In 2021	223	3.76 ± 1.16	1.42–7	0.85	0.90		0.57
	BLUE ^g^	257	3.80 ± 0.94	1.84–6.91	0.77	0.97	0.44	0.71

^a^ Mean ± SD indicates mean ± standard deviation. ^b^ Range indicates the disease index scale of the FER. ^c^ *σ_g_*^2^ indicates the genetic variance. ^d^ *σ_e_*^2^ indicates the error variance. ^e^ *σ_ge_*^2^ indicates the genotype–environment interactions variance. ^f^ *H*^2^ indicates the broad-sense heritability. ^g^ Combined indicates BLUE (the best linear unbiased estimates) value.

**Table 2:** Detailed information on the five quantitative trait loci (QTL) identified.

Chromosome	Position	LeftMarker	RightMarker	LOD ^a^	PVE ^b^ (%)	Add ^c^
*qFER1*	264	1_225878653	1_226174289	4.73	6.40	−0.22
*qFER3*	126	3_166389393	3_166460736	5.15	7.28	−0.24
*qFER5-1*	107	5_65960124	5_66200661	4.76	6.48	−0.22
*qFER5-2*	188	5_185574073	5_185568562	9.36	13.13	−0.31
*qFER7*	155	7_149858610	7_150217638	3.08	4.38	−0.18

^a^ Log-likelihood (LOD) values for additive genes were calculated using inclusive composite interval mapping (ICIM) based on multi-environment trial data. The LOD threshold used for declaring putative QTL was defined as 2.5. ^b^ Phenotypic variation explained by QTL. ^c^ A negative value indicates that the BT parent contributes to resistance, while a positive value indicates that the Xi502 parent contributes to resistance.

## Data Availability

Data are contained within the article and Supplementary Materials.

## Supplementary Materials

The following supporting information can be downloaded at: https://www.mdpi.com/article/10.3390/plants15152361/s1, Table S1. Information on the 257 inbred lines used in this study. Table S2. The list of maker information of the linkage map. Table S3. Primers used in the study.
