# Supplementary material for: Linkage Mapping Study Reveals Conservative QTL and Candidate Genes for Fusarium Ear Rot Resistance in Maize
Source: Plants (Basel). 2026 Jul 31;15(15):2361. doi: 10.3390/plants15152361 (PMC13468209; doi:10.3390/plants15152361)
Supplement: Supplementary file 1 [file plants-15-02361-s001.zip › plants-4439199-supplementary.pdf]

**Supplementary Table S1. Information of the 257 inbred lines used in this study.**

| Line name | Disease index of FER[a] | Disease index of FER[b] | Disease index of FER[c] | Blue[d] |
|---|---|---|---|---|
| 2515 | 2.125 | 3.7063 | NA | 3.3311 |
| 2538 | 6 | 5.4285 | 6 | 5.6752 |
| 2540 | 3.5666 | 5.2214 | 6.3 | 5.2035 |
| 2544 | 5.8333 | 3.7322 | 5.3333 | 4.5116 |
| 2546 | 4 | 3.3285 | 3.6667 | 3.5334 |
| 2552 | 2.2 | 2.6106 | 3.5 | 2.7645 |
| 2558 | 1.75 | 2.4 | 2 | 2.1832 |
| 2560 | 4.125 | 4 | 5.9166 | 4.5103 |
| 2563 | 2.875 | 4.4841 | 3.25 | 3.8852 |
| 2569 | 5.9 | 3.5268 | 4 | 4.0671 |
| 2570 | 3.625 | 5.6875 | 3 | 4.6382 |
| 2575 | 2.875 | 3.3 | NA | 3.1991 |
| 2576 | 6.125 | 4 | 5 | 4.6306 |
| 2578 | NA | 2.425 | 2.5 | 2.4482 |
| 2586 | 4.5 | 4.45 | NA | 4.4619 |
| 2589 | 5.3334 | 6.4514 | 4.9584 | 5.8733 |
| 2596 | 2.6667 | 4.8833 | 3.3333 | 4.0965 |
| 2597 | 2 | 3.6023 | 4.375 | 3.5157 |
| 2599 | 1.2666 | 2.6667 | 3.6667 | 2.6738 |
| 2604 | 2 | 4.9 | 3 | 3.9031 |
| 2606 | 1.25 | 4.375 | NA | 3.6334 |
| 2612 | 3.5 | 3 | NA | 3.1187 |
| 2613 | 4.1 | 3.7222 | 2.3333 | 3.4352 |
| 2616 | 4.6 | 4.6 | 4.4 | 4.5491 |
| 2619 | 5.9584 | 5.3928 | 3 | 4.8834 |
| 2620 | 3.4 | 2.909 | 3.2262 | 3.0767 |
| 2621 | 5.7 | 4.0486 | 5.9166 | 4.8165 |
| 2623 | 2.25 | 3.625 | 3.5 | 3.35 |
| 2627 | 5.4667 | 5.2 | 4.3572 | 5.0325 |
| 2647 | 1.5 | 3.5 | 3.7 | 3.1972 |
| 2649 | 5.4166 | 5.3333 | 3.8667 | 4.9745 |
| 2656 | 3.0625 | 5.475 | 3.5 | 4.5452 |
| 2701 | NA | 3.5 | NA | 3.5 |
| 2732 | 2 | 4.625 | 2.9167 | 3.7256 |
| 2739 | 5 | 5.1607 | 3.6667 | 4.7517 |
| 2742 | 4.3333 | 2.625 | 4.1666 | 3.3198 |
| 2761 | 1.55 | 3.4286 | 2.875 | 2.9553 |
| 2767 | 1.8428 | 2.5128 | 1.6667 | 2.1788 |
| 2788 | 2 | 2.725 | 3.0833 | 2.6881 |
| 2508 | NA | 2.375 | 2.75 | 2.491 |
| 2516 | 3.125 | 3.6742 | 3.5 | 3.5327 |
| 2520 | 3.375 | 3.625 | 2.3333 | 3.2518 |
| 2526 | 1.5 | 3.6964 | 2.8 | 3.0796 |
| 2535 | 2.2 | 3.0402 | 3.1111 | 2.9097 |
| 2542 | 2.75 | 4.025 | 4.0635 | 3.8093 |
| 2565 | 5.75 | 5.2678 | 5 | 5.2849 |
| 2593 | 6.8334 | 6.75 | NA | 6.7698 |
| 2594 | 3.425 | 2.9506 | NA | 3.0631 |
| 2600 | 4.0375 | 3.1515 | 5 | 3.7791 |
| 2601 | 1.625 | 2.6667 | 1.9583 | 2.302 |
| 2603 | 3.3333 | 3.3928 | 6 | 4.0464 |
| 2605 | NA | 3 | 4.2 | 3.3713 |
| 2608 | 4.1333 | 3.8012 | 5.1905 | 4.2139 |
| 2609 | 2.5 | 2.8333 | 3.75 | 3.0079 |
| 2610 | 1.6 | 2.75 | 3.25 | 2.674 |

| | | | | |
|---|---|---|---|---|
| 2611 | 3 | 4.6875 | 3 | 3.9592 |
| 2618 | 4.7 | 4.2875 | 6.1 | 4.8221 |
| 2629 | 1 | 1.8333 | 2.4375 | 1.8399 |
| 2631 | 2.5 | 6.5 | NA | 5.5508 |
| 2632 | 1.625 | 2.5347 | 3.2917 | 2.5666 |
| 2636 | 3.3 | 2.9571 | 3 | 3.0287 |
| 2637 | 2.65 | 3.875 | 4.5 | 3.8175 |
| 2641 | 1.125 | 2.7604 | 3.0857 | 2.554 |
| 2643 | 5 | 4.9111 | 3.375 | 4.5355 |
| 2645 | 1.9 | 4.2136 | 3.1111 | 3.5236 |
| 2648 | NA | 3.25 | 4 | 3.4821 |
| 2650 | 2.125 | 2.7889 | 3.75 | 2.9163 |
| 2657 | 2 | 3.2857 | NA | 2.9806 |
| 2670 | 4.2 | 5.3334 | 4.6667 | 4.9631 |
| 2688 | 2.5 | 5.8194 | 3.6 | 4.667 |
| 2689 | 5 | 5.85 | 4.75 | 5.4195 |
| 2715 | 1.8 | 3.3095 | NA | 2.9513 |
| 2719 | 2.1 | 3.0833 | 3.3333 | 2.9731 |
| 2721 | 3.25 | 4.2841 | 4.4 | 4.1307 |
| 2728 | 1.875 | 3.5714 | 3.0714 | 3.144 |
| 2741 | 5 | 7 | 4.1666 | 5.9246 |
| 2743 | 1.5 | 3.5318 | 3.4127 | 3.1421 |
| 2744 | 2.6 | 4.9762 | 4.35 | 4.3964 |
| 2748 | 6.25 | 3.3636 | 4.6125 | 4.1922 |
| 2750 | 1.3333 | 4.3125 | 3.8571 | 3.6696 |
| 2753 | 2.6667 | 2.1818 | 4.0682 | 2.7481 |
| 2755 | 4.5 | 3.5625 | NA | 3.785 |
| 2756 | 2.875 | 5.0139 | 3.75 | 4.3137 |
| 2759 | 4.5 | 4 | 3.4 | 3.9356 |
| 2768 | 2 | 6.3334 | 3.1667 | 4.7603 |
| 2769 | 2.7083 | 3.2143 | 2.7778 | 3.0136 |
| 2773 | 3.8833 | 3.5764 | 3.4643 | 3.6021 |
| 2774 | 1.85 | 2.3125 | 3.1428 | 2.4422 |
| 2777 | 3.525 | 2.75 | 3.0833 | 2.972 |
| 2778 | 7 | 5 | NA | 5.4746 |
| 2781 | 6.1666 | 6.05 | NA | 6.0777 |
| 2786 | 1.6667 | 3.2232 | 2.6389 | 2.7991 |
| 2787 | 1.625 | 3.2622 | 3.5 | 3.0332 |
| 2789 | 6.25 | 3.8095 | 4.9107 | 4.5216 |
| 2793 | 1.875 | 3.5278 | 2 | 2.8463 |
| 2796 | 3 | 3.8864 | 2.625 | 3.4083 |
| 2799 | 4.25 | 4.3334 | 6.5 | 4.8705 |
| 2803 | 3.75 | 5.5238 | 3 | 4.5672 |
| 2804 | 3.8333 | NA | 2.625 | 3.1202 |
| 2813 | 2 | 2.3125 | 2.5 | 2.305 |
| 2821 | 6.3 | 6.1609 | 5.5 | 6.0171 |
| 2824 | 2.5417 | 2.25 | 3.8333 | 2.7049 |
| 2825 | 4.8334 | 4.625 | 6.1 | 5.0376 |
| 2828 | 3.7666 | 4.5357 | 3.6 | 4.1613 |
| 2830 | 3.5 | 2.9127 | 3 | 3.0388 |
| 2833 | 1.8333 | 2.6357 | 2.75 | 2.5229 |
| 2835 | 3.125 | 4.3334 | 4 | 4.0347 |
| 2518 | 2.1428 | 4.2916 | 2.3333 | 3.4128 |
| 2551 | 5.7 | 5.0416 | 3.75 | 4.8291 |
| 2574 | NA | 2.7322 | 5.6666 | 3.6402 |
| 2598 | 1.25 | 3.8542 | 3.1191 | 3.2063 |
| 2772 | 3.8 | 3.4833 | 3.5 | 3.5436 |
| 2501 | 4 | 3.2159 | 3.8333 | 3.5119 |

| | | | | |
|---|---|---|---|---|
| 2502 | 3.4 | 3.2143 | 4 | 3.4473 |
| 2505 | 3.175 | 3.5666 | 4.5 | 3.7351 |
| 2506 | 2.75 | 3.7273 | 4.1 | 3.6494 |
| 2509 | 2 | 3.3016 | 2 | 2.7398 |
| 2510 | 1.8333 | 2.8117 | NA | 2.5795 |
| 2524 | 2.6 | 3.1667 | 1.9375 | 2.7533 |
| 2525 | 1.8667 | 3.1 | 3.6428 | 3.0201 |
| 2527 | 4 | 5 | 3.9167 | 4.5472 |
| 2528 | 5.7667 | 3.8572 | 3.1905 | 4.0251 |
| 2529 | 3.0167 | 3.5208 | 3.75 | 3.49 |
| 2530 | 3.2 | 4.3333 | 2.75 | 3.7296 |
| 2531 | 3.6 | 3.3409 | 4.1666 | 3.5971 |
| 2534 | 5.5 | 5.65 | 3 | 4.9485 |
| 2539 | 5.5 | 4.5 | NA | 4.7373 |
| 2547 | 3.5 | 3.9909 | 4.4667 | 4.0253 |
| 2548 | 4.25 | 4.2857 | 2.25 | 3.7608 |
| 2549 | 2.5 | 2.4 | 3 | 2.5705 |
| 2553 | 3.2167 | 4.3056 | 3.9167 | 4.0139 |
| 2556 | 2.1667 | 2.5833 | 3 | 2.6158 |
| 2557 | 4 | 4 | 1.6667 | 3.4057 |
| 2559 | 3.375 | 2.6644 | NA | 2.833 |
| 2561 | 1.5417 | 1.75 | 3.8333 | 2.2438 |
| 2562 | 3 | 4.2552 | 6.6667 | 4.6475 |
| 2564 | 1.2917 | 2.1667 | 7 | 3.2431 |
| 2566 | 2.4167 | 3.5 | 3.75 | 3.3721 |
| 2571 | 1.9 | 2.5272 | NA | 2.3784 |
| 2573 | 4.1666 | 3.7 | 4.5 | 3.9863 |
| 2579 | NA | 4.6 | 4 | 4.4143 |
| 2580 | 3.625 | 3.2708 | 4.75 | 3.7103 |
| 2581 | 2.8333 | 3.8714 | NA | 3.6251 |
| 2583 | 3 | 3.375 | NA | 3.286 |
| 2588 | 2.9334 | 3.3889 | 5.25 | 3.7824 |
| 2590 | 2.8333 | 5.5 | NA | 4.8672 |
| 2592 | 6.6666 | 5.8571 | NA | 6.0492 |
| 2595 | 4.3215 | 3.1222 | 4.5 | 3.6853 |
| 2633 | 3.325 | 3.6 | 4.2 | 3.7042 |
| 2634 | 2.5 | NA | 3.8 | 3.2673 |
| 2635 | 3 | 4.625 | 5.125 | 4.465 |
| 2640 | 3.1667 | 6.5396 | 3.6309 | 5.2022 |
| 2642 | 6.15 | 5.8428 | 3.8809 | 5.3974 |
| 2644 | 2.9583 | 2.7119 | 2.25 | 2.6378 |
| 2651 | 4.1666 | 3.1667 | 4.25 | 3.6195 |
| 2652 | 2.5833 | 4.0834 | 3.0833 | 3.5633 |
| 2654 | 1.25 | 3.25 | 4.5834 | 3.2359 |
| 2655 | 4.5 | 3.5 | 4 | 3.8042 |
| 2658 | 4.675 | 3.7727 | 4.6072 | 4.1449 |
| 2660 | 5.75 | 5.9584 | 2 | 4.9132 |
| 2662 | 4.5 | 3.6 | 3 | 3.6063 |
| 2664 | 1.9 | 3.9444 | 2.3125 | 3.1672 |
| 2665 | 4.4 | 4.9445 | 3.225 | 4.4102 |
| 2666 | 1.925 | 2.7222 | 3.4 | 2.7539 |
| 2668 | 4.5 | 4.475 | 3.925 | 4.3393 |
| 2669 | 1.2 | 3.5 | 4.1667 | 3.263 |
| 2671 | 2.5 | 4.6714 | 4.3334 | 4.2013 |
| 2672 | 3.8333 | 5.225 | NA | 4.8947 |
| 2673 | 4.25 | 4.5793 | 3.6905 | 4.2947 |
| 2674 | 3.4167 | 3.1458 | 1.4167 | 2.7533 |
| 2676 | 1.6667 | 5.865 | 2 | 4.138 |

| | | | | |
|---|---|---|---|---|
| 2677 | 6.5 | 7 | 7 | 6.9116 |
| 2678 | 3.9 | 2.6616 | 3.1428 | 3.0032 |
| 2679 | 2.75 | 2.6428 | 3.1 | 2.7782 |
| 2680 | 2 | 2.7 | 3 | 2.6526 |
| 2682 | 4.2 | 4.909 | 1.6667 | 3.9577 |
| 2683 | 2.2917 | 5.675 | 2.4286 | 4.2497 |
| 2685 | 1.3333 | 3.5903 | 3.5 | 3.1681 |
| 2686 | 2 | 3.5625 | 3 | 3.1429 |
| 2687 | 2 | 4.0625 | 4 | 3.6818 |
| 2690 | 2.9167 | 4.4524 | NA | 4.0879 |
| 2691 | 2.8 | NA | 6 | 4.6886 |
| 2692 | 2.25 | 4.4286 | 3.8333 | 3.8917 |
| 2693 | 3 | 4.5 | 3 | 3.8526 |
| 2694 | 6 | 4.0278 | NA | 4.4958 |
| 2695 | 2.25 | 2.7381 | 5.2 | 3.2789 |
| 2699 | 2.8333 | 3 | 3.6667 | 3.1403 |
| 2700 | 2.2 | 2.2857 | 5.3334 | 3.0468 |
| 2702 | 2.1667 | 3.8333 | 6.5 | 4.2178 |
| 2703 | 3.6 | 4.6666 | 3.35 | 4.1426 |
| 2704 | 2.25 | 3.5 | 3 | 3.1516 |
| 2705 | 3.6667 | 4.4571 | NA | 4.2696 |
| 2706 | 1.8 | 2.3961 | 2.5238 | 2.3232 |
| 2707 | 1.6667 | 3.5 | 2.4107 | 2.8983 |
| 2708 | 2.6667 | 2.4508 | 2.6889 | 2.5496 |
| 2711 | 3.125 | 6.0625 | 4.3 | 5.094 |
| 2716 | 3.0417 | 4.697 | 2.9583 | 3.9614 |
| 2717 | 1.9 | 3.9286 | 2.6667 | 3.2484 |
| 2718 | 5.8334 | 5.9584 | 3 | 5.1827 |
| 2720 | 2 | 4.1857 | 3.3333 | 3.582 |
| 2722 | 2 | 2.125 | 2 | 2.0711 |
| 2724 | 1 | 3.5 | 3.25 | 2.9942 |
| 2726 | 1.9167 | 3.8889 | 4.25 | 3.6321 |
| 2727 | 1 | 2.7053 | NA | 2.3007 |
| 2729 | 3 | 4.3472 | 4.1666 | 4.0629 |
| 2730 | 3.8 | 3.9444 | 5 | 4.1878 |
| 2733 | 2.25 | 3.7 | 3.5 | 3.3926 |
| 2735 | 1 | 4.3435 | 2.75 | 3.3462 |
| 2736 | 2.4 | 3.5546 | 2.6667 | 3.1242 |
| 2737 | 2.75 | 6.1875 | 5 | 5.2771 |
| 2745 | 1.5 | 5.6072 | 5 | 4.7261 |
| 2746 | 7 | 3.425 | 4.5 | 4.3311 |
| 2747 | 5.875 | 6.1333 | NA | 6.072 |
| 2749 | 1.6667 | 3.5549 | NA | 3.1068 |
| 2754 | 1.6667 | 3.5 | 2.5 | 2.921 |
| 2757 | 2 | 3.9167 | 2.5333 | 3.2253 |
| 2758 | 1.8 | 3.7 | 3.6428 | 3.3494 |
| 2763 | 1 | 5.3928 | 4.1667 | 4.3036 |
| 2766 | 1.5 | 3.6111 | 2.0833 | 2.8486 |
| 2770 | NA | 3.8 | 3.5 | 3.7072 |
| 2771 | 4 | 3 | 3.4 | 3.2788 |
| 2775 | 4.4 | 4.9091 | 4.1072 | 4.6148 |
| 2776 | 1.5 | 2.9722 | 2.4583 | 2.5809 |
| 2779 | 3.9167 | 5.2008 | 3.925 | 4.6487 |
| 2780 | 1.9286 | 2.5833 | 3 | 2.5737 |
| 2782 | 3.0417 | 3.7727 | 3.3375 | 3.5325 |
| 2783 | 3.75 | 5.3667 | 4.25 | 4.7963 |
| 2784 | 3 | 3.1445 | 3.625 | 3.2413 |
| 2785 | 2.125 | 2.1786 | 3.25 | 2.442 |

| | | | | |
|---|---|---|---|---|
| 2790 | 2.2667 | 3.75 | 3.0833 | 3.3178 |
| 2791 | 3.5 | 4.0286 | NA | 3.9031 |
| 2794 | 4.0667 | 4.4285 | 2.6905 | 3.9218 |
| 2797 | 3.6 | 3.4286 | 6 | 4.1139 |
| 2798 | 2.7 | 5.2857 | 3.75 | 4.4372 |
| 2800 | 3 | 4.5793 | 5.5 | 4.5345 |
| 2801 | 5.75 | 6.8182 | NA | 6.5647 |
| 2802 | 3.4167 | 4.5 | 3 | 3.9263 |
| 2805 | 3.975 | 3.9167 | 2.875 | 3.6617 |
| 2806 | 3.9167 | 4.4167 | 3.3333 | 4.0523 |
| 2807 | 5.5 | 3.75 | 7 | 4.8874 |
| 2808 | 3.0833 | 3.8125 | 2 | 3.2219 |
| 2809 | 2.5333 | 4.275 | NA | 3.8617 |
| 2810 | 6.8334 | 5 | 6 | 5.579 |
| 2811 | 2.4822 | 3.9286 | 3 | 3.4362 |
| 2812 | 2.9167 | 3.3333 | 2.125 | 2.9519 |
| 2814 | 2.7917 | 3.9911 | 4.5 | 3.9086 |
| 2815 | 3.1 | 3.6191 | 5 | 3.879 |
| 2816 | 6.2 | 4.7636 | 7 | 5.5873 |
| 2817 | 5.15 | 4.6919 | NA | 4.8006 |
| 2818 | 3.25 | 4.9732 | 4.5 | 4.5479 |
| 2822 | 5.9285 | 4.6262 | 6.25 | 5.2702 |
| 2823 | 3 | 2.1556 | NA | 2.3559 |
| 2826 | 4.0834 | 4.5 | 6.0834 | 4.8296 |
| 2827 | 5.075 | 4.25 | 4 | 4.3322 |
| 2829 | 4 | 4.0278 | 4.7667 | 4.2111 |
| 2831 | 3.025 | 3.9716 | 3.8333 | 3.769 |
| 2832 | 3.25 | 2.6667 | 3.6667 | 3.0246 |

[a]FER (Fusarium verticillioides ear rot) was evaluated in Zhengzhou in 2019.

[b]FER (Fusarium verticillioides ear rot) was evaluated in Xunxian in 2020.

[c]FER (Fusarium verticillioides ear rot) was evaluated in Jiaozuo in 2021.

[d]The disease index was the BLUE (The best linear unbiased estimates) value of three locations.

**Supplementary Table S2. Maker Information of the linkage map.**

| Chromosome | Markers number | Total length（cM） | Average distance between makers （cM） |
|---|---|---|---|
| 1 | 1048 | 376.88 | 0.36 |
| 2 | 737 | 281.64 | 0.38 |
| 3 | 697 | 244.51 | 0.35 |
| 4 | 730 | 240.89 | 0.33 |
| 5 | 803 | 279.35 | 0.35 |
| 6 | 568 | 218.55 | 0.38 |
| 7 | 523 | 234.44 | 0.45 |
| 8 | 691 | 273.68 | 0.40 |
| 9 | 529 | 225.42 | 0.43 |
| 10 | 481 | 165.51 | 0.34 |
| Total | 6807 | 2540.87 | 0.37 |

**Supplementary Table S3. Primers used in the study.**

| Gene | | Primer sequence (5'-3') |
|---|---|---|
| *EF1a* | Sense | TGGGCCTACTGGTCTTACTACTGA |
| | Anti-sense | ACATACCCACGCTTCAGATCCT |
| *qGRMZM2G094563* | Sense | GTACCTCCAGGAGCGCGTAAAG |
| | Anti-sense | CCTTGCCGTCGGTCTTGGAG |
| *UTRGRMZM2G094563* | Sense | GAACGACATTCTTCGCCTACG |
| | Anti-sense | GAAATGGTGCTGCGAGATAAC |
| *qGRMZM2G094602* | Sense | GGCAAGTCCTCCAAGAGAATG |
| | Anti-sense | GGGCAATCGAGCTTGGTTTAC |
| *exon1GRMZM2G094602* | Sense | ATTCCTCCAACAATCCAAGGTTC |
| | Anti-sense | ACCTTAACTCGGAAATGACGGAA |
| *exon4GRMZM2G094602* | Sense | CGGCAGGAATTACTTTCCGATACA |
| | Anti-sense | ATGCTGAATTGCTCAGTTGC |
